# Creatinine-Based Renal Function Estimates and Dosage of Postoperative Pain Management for Elderly Acute Hip Fracture Patients

**DOI:** 10.3390/ph11030088

**Published:** 2018-09-18

**Authors:** Morten Baltzer Houlind, Kristian Kjær Petersen, Henrik Palm, Lillian Mørch Jørgensen, Mia Aakjær, Lona Louring Christrup, Janne Petersen, Ove Andersen, Charlotte Treldal

**Affiliations:** 1 Optimed, Clinical Research Centre, Copenhagen University Hospital Hvidovre, Kettegård Alle 30, Department 056, 2650 Hvidovre, Denmark; lillian.moerch.joergensen@regionh.dk (L.M.J.); mia.aakjaer@sund.ku.dk (M.A.); petersen.janne@gmail.com (J.P.); ove.andersen@regionh.dk (O.A.); charlotte.treldal.02@regionh.dk (C.T.); 2 The Capital Region Pharmacy, Marielundvej 25, 2730 Herlev, Denmark; 3 Section of Pharmacotherapy, Department of Drug Design and Pharmacology, University of Copenhagen, Universitetsparken 2, 2100 København Ø, Denmark; llc@sund.ku.dk; 4 Center for Sensory-Motor Interaction (SMI), Department of Health Science and Technology, Faculty of Medicine, Aalborg University, Fredrik Bajers Vej 7, building A2-206, 9220 Aalborg Ø, Denmark; kkp@hst.aau.dk; 5 Orthopedic Department, Copenhagen University Hospital Bispebjerg, Bispebjerg Bakke 23, 2400 København, Denmark; Henrik.palm@regionh.dk; 6 Section of Biostatistics, Department of Public Health, University of Copenhagen, Øster Farimagsgade 5, Enterance B, 2nd floor, 1014 København, Denmark; 7 Emergency Department, Copenhagen University Hospital Hvidovre, Kettegård Alle 30, Department 436, 2650 Hvidovre, Denmark

**Keywords:** renal function, kidney function, glomerular filtration rate, elderly, analgesic, pain management, drug dose adjustment, drug dosing, patient safety, clinical pharmacy

## Abstract

Many analgesics and their metabolites are renally excreted. The widely used Chronic Kidney Disease Epidemiology Collaboration (CKD-EPI)-estimated glomerular filtration rate (eGFR) equations are not developed for use in the elderly, while the recent Berlin Initiative Study (BIS), Full Age Spectrum (FAS), and Lund-Malmö revised (LMR) equations are. This observational study investigated differences between creatinine-based eGFR equations and how the choice of equation influences dosage of analgesics in elderly (≥70 years) patients admitted with acute hip fracture. eGFR was calculated by the CKD-EPI, BIS, Cockcroft-Gault (CG), FAS, LMR, and Modification of Diet in Renal Disease (MDRD) equations. Standard daily dose for postoperative pain medications ibuprofen, morphine and gabapentin was simulated for each equation according to dosage recommendations in Renbase^®^. For 118 patients, mean eGFR from the CKD-EPI, BIS, CG, FAS, LMR, and MDRD equations was 67.3 mL/min/1.73 m^2^, 59.1 mL/min/1.73 m^2^, 56.9 mL/min/1.73 m^2^, 60.3 mL/min/1.73 m^2^, 58.9 mL/min/1.73 m^2^, and 79.1 mL/min/1.73 m^2^, respectively (*p* < 0.0001). Mean difference to CKD-EPI was −10.4 mL/min/1.73 m^2^ to 11.8 mL/min/1.73 m^2^. Choice of eGFR equation significantly influenced the recommended dose (*p* < 0.0001). Shifting to BIS, FAS, or LMR equations led to a lower recommended dose in 20% to 31% of patients. Choice of eGFR equation significantly influenced dosing of ibuprofen, morphine, and gabapentin.

## 1. Introduction

Optimization of postoperative pain management in elderly patients is essential for fast-track surgery and patient-related outcomes [1,2]. Several medication regimens have been proposed to optimize postoperative pain recovery [3,4,5]. Unfortunately, many of these most frequently used medications and their metabolites are renally excreted and therefore a challenge to dose in patients with renal impairment [6,7]. Postoperative analgesics to patients with hip fractures, are often prescribed in standard doses, but must be adjusted according to renal function [7]. Renal function declines with age, and this decline is further accelerated by co-morbidities such as hypertension, diabetes mellitus, and chronic inflammation [8,9,10]. Because of their high age, high comorbidity and reduced kidney function, most acute hip fracture patients are categorized as fragile patients [11,12,13,14,15,16]. Elderly patients are also more likely to experience unpredictable pharmacokinetic and pharmacodynamic variations, making them more susceptible to dosing errors [17]. Several renally excreted analgesics in particular are known to cause adverse drug reactions when they or their active metabolites accumulate in the body or to cause nephrotoxicity [6,7,18]. These so-called “renal risk medications” include the first line choices in postoperative pain management, such as ibuprofen, morphine, and gabapentin. Ibuprofen is, like other Nonsteroidal Anti-Inflammatory Drugs (NSAIDs), nephrotoxic [19]. Morphine is metabolized to morphine-3-glucuronide and morphine-6-glucuronide, which are both excreted renally [18]. Gabapentin is almost exclusively eliminated unchanged by renal excretion [20]. The adverse reactions of ibuprofen, morphine, and gabapentin are dose-dependent, stressing the importance of prescribing these drugs at doses individually adjusted for the actual kidney function [6,7].

Glomerular filtration rate (GFR) is considered the best indicator of renal function and is commonly used to guide optimal dosing of medications [21]. Exogenous gold standard markers such as inulin and iohexol give accurate measures of GFR, but these are practically infeasible and too expensive to routinely use in hospital settings [22,23]. Instead, GFR is typically estimated based on serum concentration of an endogenous biomarker such as creatinine, which is fast and cheap to obtain [23]. The Chronic Kidney Disease Epidemiology Collaboration (CKD-EPI) equation based on creatinine [24] is commonly used in clinical practice worldwide and recommended both by “Kidney Disease: Improving Global Outcomes” (KDIGO) [25] and the Danish Society of Nephrology. Other widely used estimated GFR (eGFR) equations include the Modification of Diet in Renal Disease (MDRD) [26] and Cockcroft-Gault (CG) [27] equations. Unfortunately, the CKD-EPI and MDRD equations from North America are not developed for use among patients ≥70 years and are known to overestimate GFR in these patients [28,29,30]. Conversely, it is well known that the CG equation systematically underestimates GFR [31,32], but the equation is often used in drug development studies. The more recently developed European eGFR equations, including the Berlin Initiative Study (BIS) [29], Full Age Spectrum (FAS) [33], and Lund-Malmö revised (LMR) [34] equations, were adapted to more accurately estimate GFR in the elderly. However, so far, no studies have been conducted to directly compare how these equations perform in elderly hip fracture patients, and there is little research discussing the implications of switching between equations for dosing of analgesics. The aims of this study are: (1) to compare renal function estimates and CKD classification between the BIS, CG, FAS, LMR, and MDRD equations and the standard CKD-EPI equation in elderly hip fracture patients receiving postoperative pain management, and (2) to demonstrate how choice of eGFR equation influences dose recommendations for ibuprofen, morphine, and gabapentin.

## 2. Results

One hundred and eighty-three patients were hospitalized with acute hip fracture during the study period. Patients were excluded due to <70 years (n = 52), potential AKI on third postoperative day (n = 6), or death before the third postoperative day (n = 7). Patient characteristics for included patients (n = 118) are shown in Table 1. Among included patients, 68% were female and the median age was 82.6 years.

### 2.1. Estimated Glomerular Filtration Rate

Mean eGFR from the CKD-EPI, BIS, CG, FAS, LMR, and MDRD equations are given in Table 2. Mixed models with renal function estimate values showed that the BIS, CG, FAS, LMR, and MDRD equations were all significantly different from CKD-EPI (*p* < 0.0001). The BIS, CG, FAS, and LMR equations yield significantly lower eGFR than CKD-EPI, with a mean difference ranging from −7.0 mL/min/1.73 m^2^ to −10.4 mL/min/1.73 m^2^. The MDRD equation yields significantly higher eGFR than CKD-EPI, with a mean difference of 11.8 mL/min/1.73 m^2^ (*p* < 0.0001). 

No differences in eGFR were found by comparison of the BIS, FAS and LMR equations (*p* ≥ 0.142). The CG equation yields significantly lower eGFR than all other equations, with a mean difference of −2.0 mL/min/1.73 m^2^ to −22.2 mL/min/1.73 m^2^ *p* ≤ 0.030). Finally, the MDRD equation yields an eGFR significantly higher than all other equations, with a mean difference of 11.8 mL/min/1.73 m^2^ to 22.2 mL/min/1.73 m^2^ (*p* < 0.0001).

### 2.2. CKD Re-Classification Compared with the CKD-EPI

The distributions of CKD stages based on the eGFR equations are shown in Table 3. The CKD-EPI equation classified 79 patients (66.9%) in CKD stages 1–2, and only 39 patients (n = 33.1) in CKD stages 3–5. The BIS, FAS, and LMR equations classified between 53 patients and 59 patients (44.9% to 50.0%) in CKD stages 1–2, and between 59 patients and 65 patients (50.0% to 55.1%) in CKD stages 3–5. The CG equation showed similar classification patterns as BIS, FAS, and LMR, but 70 patients (59.3%) were classified in CKD stages 3–5. In contrast, the MDRD equation only classified 32 patients (27.1%) in CKD stages 3–5. Table 4 shows the agreement of CKD classification between the eGFR equations. The MDRD equation had the highest agreement with CKD-EPI (к = 0.70), while the CG equation had the lowest agreement (к = 0.57). The BIS, FAS, and LMR equations all had almost perfect agreement with each other (к ≥ 0.85).

### 2.3. Shift in Recommended Prescription Dose of Ibuprofen, Morphine, and Gabapentin

**Figure 1:**
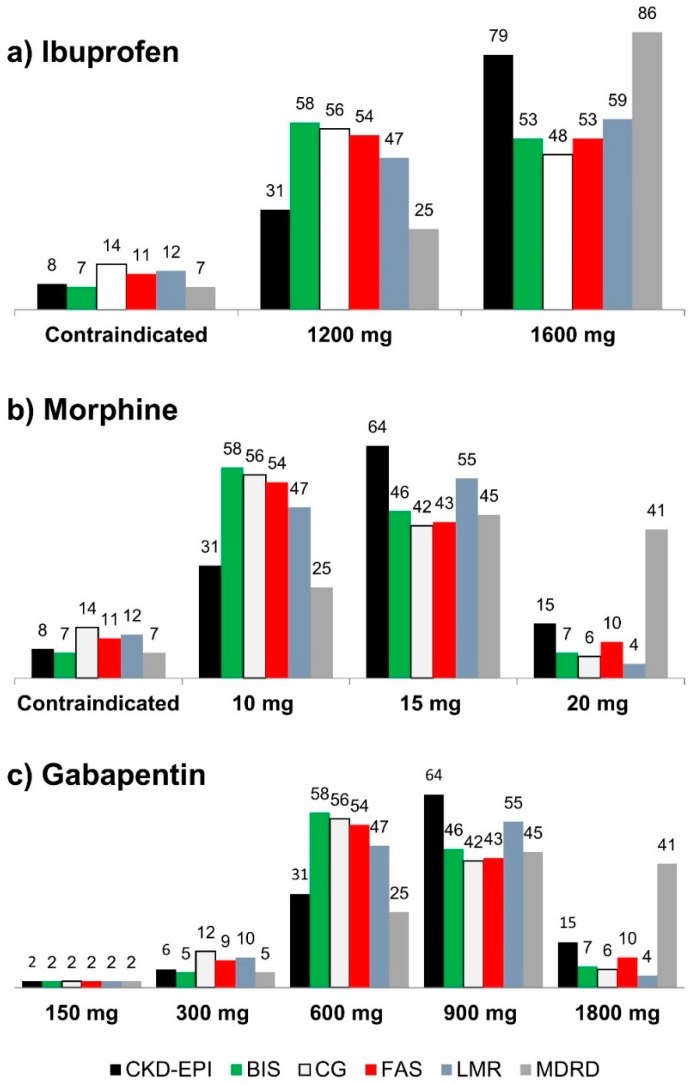
(**a**–**c**) Simulated recommended doses of ibuprofen (**a**), morphine (**b**), and gabapentin (**c**) according to the six different creatinine-based eGFR equations (n = 118). *CKD-EPI* Chronic Kidney Disease Epidemiology Collaboration, *BIS* Berlin Initiative Study, *CG* Cockcroft-Gault, *FAS* Full Age Spectrum, *LMR* Lund-Malmö revised, *MDRD* Modification of Diet in Renal Disease

Figure 1 and Table 5 show the potential changes in doses for ibuprofen, morphine, and gabapentin when switching between the CKD-EP, BIS, CG, FAS, LMR, or MDRD equations. Recommended doses for all three analgesics were statistically significantly different with CKD-EPI compared to the other eGFR equations (*p* ≤ 0.0078). No differences in recommended ibuprofen and morphine doses were found between BIS, FAS and LMR equations (*p* ≥ 0.125). However, gabapentin dose recommendations were significantly lower with the LMR equation compared to the BIS and FAS equations (*p* ≤ 0.0001). All recommended doses for all three analgesics were statistically significantly lower with CG compared to the other eGFR equations (*p* ≤ 0.0287), except for gabapentin based on the LMR equation (*p* = 0.1082). Finally, all recommended doses for all three analgesics were statistically significantly higher with MDRD compared to the other eGFR equations (*p* ≤ 0.0078).

Overall, shifting from CKD-EPI to BIS, FAS, or LMR would result in a lower recommended dose of gabapentin and morphine for 34 to 35 patients (29% to 30%) and 24 to 29 patients (20% to 25%) for ibuprofen (Table 5). Recommended doses for ibuprofen, morphine, and gabapentin would be reduced according to renal function in over half of patients by using BIS, CG, FAS, or LMR, while they would be reduced in only one quarter of patients when using CKD-EPI or MDRD. Furthermore, ibuprofen and morphine would be contraindicated in about twice as many patients by using CG, FAS, or LMR instead of CKD-EPI, BIS, or MDRD.

## 3. Discussion

In the current study, estimates of renal function obtained with six equations were compared and the impact of their use for postoperative pain management in elderly hip fracture patients was assessed. It was found that the CG, BIS, FAS, and LMR equations estimated GFR significantly lower than CKD-EPI, while the MDRD equation estimated GFR significantly higher. These differences between GFR estimates based on the six equations led to significant differences in standard dosing of ibuprofen, morphine, and gabapentin according to renal function in 20–31% in elderly hip fracture patients.

### 3.1. eGFR Equations Based on Creatinine and Elderly

As expected, the recently developed BIS, FAS and LMR equations estimated GFR lower than CKD-EPI and classified considerably more patients in CKD stage III or below (<60 mL/min/1.73 m^2^) [29,33,34]. The CKD-EPI equation based on creatinine is recommended by KDIGO [25] and used internationally in clinical practice. However, there are several drawbacks to using this equation in elderly acutely hospitalized patients. First, the CKD-EPI equation was not developed to estimate GFR in the elderly; rather, it developed to improve GFR estimation >60 mL/min/1.73 m^2^ in adults [24], but optimization of prescription is primarily relevant at low renal function (<60 mL/min/1.73 m^2^). A possible explanation for CKD-EPI performing poorly in elderly patients with low renal function is that the cohort in which CKD-EPI was developed only contained 4% (n = 217) over 70 years [24]. The more recent BIS, LMR and FAS equations were, however, developed in populations with a higher percentage of elderly patients. BIS was developed in a cohort where all patients were above 70 years (n = 610) [29]. LMR included 27% (n = 230) of patients over 70 years and was developed with the explicit goal of improving eGFR at lower levels of renal function [34]. FAS included 26% (n = 1764) patients over 70 years and was developed based on average GFR and age-normalized serum creatinine [33].

Recent studies have found that the BIS, FAS and LMR equations based on creatinine have a higher percentage of estimates within 30% of the measured GFR (P30 accuracy) than CKD-EPI in elderly patients [35,36,37]. However, there is no consensus about which of these alternative equations is best. The MDRD equation overestimates GFR in the elderly [28,29,30]. Heldal et al. found that BIS, CG, FAS, and LMR were more accurate than CKD-EPI in stable elderly kidney transplant patients [38], while results from several other studies have proven that CG underestimates GFR in the elderly [31,32]. The BIS equation was developed specifically in elderly patients and seems to be the most promising alternative to CKD-EPI. An in-depth review supports that BIS is most accurate in patients with GFR < 60 mL/min/1.73 m^2^ [39], and a direct comparison by Oscanoa et al. of BIS and CKD-EPI in elderly patients suggests that BIS is more accurate [40]. However, no studies have directly compared these creatinine-based equations among elderly acutely hospitalized patients. The findings in the current study highlight the high degree of variability among the equations and emphasize the importance of considering how this variability could affect prescribing of renal risk medications.

A general challenge of using creatinine to estimate renal function in elderly patients is the biomarker’s dependence on age, sex, race, muscle mass, and nutritional status [41,42]. The eGFR equations try to account for age, sex, and race. However, the association to muscle mass is particularly problematic in patients with low muscle mass, such as elderly, who will tend to have low baseline creatinine production. Segarra et al. studied the accuracy of CKD-EPI in hospitalized patients and found that CKD-EPI overestimates eGFR with a median bias of 2.7 mL/min/1.73 m^2^ in patients over 70 years and 5.9 mL/min/1.73 m^2^ in malnourished patients [43]. Median BMI among our acute hip fracture patients was only 22.4%, and 12% of the patients were underweight (BMI ≤ 18.5). Acute hip fracture patients are likely to be even more fragile than patients in the Segarra et al. cohort, so it is reasonable to expect that CKD-EPI also over-estimates eGFR in the current patient cohort presented here. Future studies should investigate which eGFR biomarker(s) and equation are most accurate for elderly frail hospitalized patients. These types of studies are needed to perform accurate pharmacokinetic studies in the elderly in drug development and for optimizing medication prescribing in the clinic

### 3.2. Renal Risk Medications and How to Meet the Challenge Clinical Practice

Inappropriate prescribing based on a patient’s renal function is a well-known challenge. A study in Sweden by Helldén et al. found that 4.7% of all acutely hospitalized elderly patients were admitted due to adverse drug reactions related to impaired renal function [44]. Furthermore, it has been reported that 14% of elderly in primary care [45,46] and 23% of elderly in the hospital [47] lack proper dose adjustment according to renal function. Since approximately 40% of all medications must be dosed according to renal function [21], this clinical challenge applies to most medications used in postoperative pain management [7]. However, few studies have investigated how choice of eGFR equation influences dose recommendations in the elderly, and most of these studies only consider high-risk medications such as novel oral anticoagulants (NOACs). Results from two European studies in elderly patients showed that use of CG compared to CKD-EPI or MDRD results in lower eGFR values and lower recommended doses of NOACs [48,49]. On the other hand, a third study from Canada found that CG gave higher eGFR values than both CKD-EPI and MDRD, although this patient cohort had a mean BMI of 28 kg/m^2^ [50]. There is evidently still debate about which eGFR equation is best among elderly patients, and our own results emphasize that choice of eGFR equation has a direct influence on medication prescribing for postoperative pain management in elderly patients.

Postoperative pain management is essential for patient quality and patient related outcomes in fast track surgery, and careful dosing is required to avoid complications and hospital readmission. Overdosing of morphine, for example, can lead to serious adverse reactions such as CNS and respiratory depression as well as narcosis [7,18]. Ibuprofen, morphine and gabapentin should all be avoided in patients with AKI due to the risk of accumulation of the substances, their metabolites and/or toxicity. To address this challenge in the clinic, dialogue with patients about their pain and medication dosing must be an integrated part of hospital ward rounds. Clinical-decision platforms or medication reviews should also be used in combination with patient dialogue to optimize prescribing practice. Lastly, clinicians treating elderly patients should consider use of pain medications with pharmacokinetic properties that make their effects less dependent on renal function. One example is oxycodone, which is metabolized in the liver to noroxycodone and oxymorphone [51], while 14% of the initial dose is excreted through the kidneys unchanged [52]. The major metabolite noroxycodone its inactive [53], and the active metabolite oxymorphone is formed only in minor amounts [51,54]. Oxymorphone is excreted mainly as the inactive oxymorphone-3-glucuronide conjugate [55]. Taken together, this makes oxycodone a safer alternative to morphine in patients with reduced renal function. Gabapentinoids is excreted unchanged renally and should be doses strictly according to the renal function [20]. Alternatively, tricyclic antidepressants are often used to treat neuropathic pain and are also independent of renal function. Unfortunately, there are no such alternatives to ibuprofen, since nephrotoxicity is a problem for the entire NSAID class [19].

### 3.3. Strengths and Limitations

The main strength of this study is that it identifies a daily clinical challenge of dosing renal risk medications in an unselected group of elderly hip fracture patients. This study also has several limitations. First, the current study lacks a gold standard for measuring GFR. Therefore, we compare the relative accuracies of each GFR equation and discuss how medication dosing would change by switching between equations. Second, this is a data simulation study and does not investigate clinical outcomes related to the simulation. Third, we simulate prescribing of NSAID to all patients in this study to show the clinical challenge of prescribing NSAID isolated to the choice of eGFR equation. In general, all NSAIDs should be used with caution in elderly patients due to the risk of ulcers, bleeding, and heart failure [56]. Some patients in this study would probably not be candidates to receive ibuprofen postoperatively in clinical practice due to co-morbidities. Fourth, we only calculate the normalization of GFR to a standardized body-surface area of 1.73 m^2^. In drug development, the US Food and Drug Administration recommends the consideration of eGFR in relative or absolute values [57], while the European Medicines Agency only recommends eGFR in absolute values [58].

However, the finding of high variability between GFR equations should serve as a caution to clinicians who rely on only one equation in daily practice. Finally, this study is limited by the use of creatinine on the third postoperative day to define potential AKI. KDIGO guidelines suggest that a follow-up creatinine measurement should be taken at a later day to confirm AKI diagnosis [59].

## 4. Materials and Methods

### 4.1. Ethics Approval

Registry-based studies do not need prior ethical approval in Denmark [60]. The study was approved by the Danish Data Protection Agency (j.no. 2014-41-3001). All data was anonymized prior to access for this study.

### 4.2. Design and Setting

This was an observational registry study performed in the acute hip fracture ward, orthopaedic department, Copenhagen University Hospital, Hvidovre, Denmark from 1 January to 1 April 2015. Inclusion criteria were: acute hip fracture. Exclusion criteria were: age below 70 years or acute kidney injury (AKI) on the third postoperative day.

In accordance with standard practice, pain management from pre-operation to the morning of fourth postoperative day consisted of epidural infusion of 4 mL/h bupivacaine, 0.125%, with 50 µg/mL morphine as well as oral paracetamol 4 g per day. The daily dose of paracetamol was reduced to 2 g in case of: mild to moderate hepatic impairment (Child-Pugh Class A or B), severe malnutrition, anorexia, BMI ≤ 18.5 kg/m^2^, chronic alcohol use or sepsis. Paracetamol was considered contraindicated in case of severe impairment (Child-Pugh Class C). During morning rounds of the third postoperative day, a pharmacological pain treatment was chosen to replace the epidural infusion. This pharmacological pain treatment consisted of ibuprofen, morphine or gabapentin, or a combination of the three in standard doses according to renal function and comobilities. Treatment was initiated immediately after the ward round to have an effect before discontinuation of epidural infusion on the following day. Oral paracetamol was continued in all patients. In this study, a suggested dose was simulated for each analgesic according to renal function on a patient-by-patient basis. The main outcome measures were GFR estimated with CKD-EPI_,_ BIS, CG, FAS, LMR, and MDRD; differences between GFR estimates by CKD-EPI and estimates by BIS, CG, FAS, LMR, and MDRD; and suggested doses for ibuprofen, morphine, and gabapentin according to each eGFR equation.

### 4.3. Study Data and Measurement

Information concerning hospital admission as well as comorbidities registered with ICD10 (International Statistical Classification of Diseases and Related Health Problems) is available in the National Patient Registry. Data concerning height and weight is available in the Danish Interdisciplinary Register for Hip Fractures. Information about dispensation of medication prescriptions prior to admission is available in The Danish Register of Medicinal Product Statistics. Finally, data about renal function is available in the Register of Laboratory Results for Research.

Serum creatinine values were available for the first, second, and third postoperative day, as well as the highest and lowest values between admission and discharge. Serum creatinine was measured at the Clinical Biochemical Department at Hvidovre University Hospital, Denmark, on a Roche Cobas^®^ c 8000 701/702 (Roche Diagnostics International Ltd., Rotkreuz ZG, Switzerland) with a module instrument using the Roche Creatinine Plus version 2 IDMS-traceable enzymatic assay (coefficient of variation 1.4%) as recommended in KDIGO 2012 Guideline [25].

eGFR was calculated using six creatinine-based equations: CKD-EPI [24], BIS [29], CG [27], FAS [33], LMR [34], and MDRD [26] (see Appendix A). All equations account for age and sex. CKD-EPI and MDRD also adjust for race [24,26], while CG adjusts for body weight [27]. All GFR estimates were calculated relative to body surface area (mL/min/1.73 m^2^), where standard body surface area (BSA) is set to 1.73 m^2^. For comparison with the other equations, creatinine clearance by CG was normalized per 1.73 m^2^ of BSA using the DeBois and DeBois equation for calculating BSA [61]. The severity of renal impairment was determined for each equation and classified according to the 2003 National Kidney Foundation Kidney Disease Outcomes Quality Initiative Classification [62]. This classification system uses the following GFR value cutoffs as prescribing guidelines for renal risk medications: “normal GFR (CKD stage 1)” (eGFR ≥ 90 mL/min/1.73 m^2^), “mildly decreased GFR (CKD stage 2)” (eGFR 60–89 mL/min/1.73 m^2^), “moderately decreased GFR (CKD stage 3)” (eGFR 30–59 mL/min/1.73 m^2^), “severely decreased GFR (CKD stage 4)” (eGFR 15–29 mL/min/1.73 m^2^), or “kidney failure (CKD stage 5)” (eGFR < 15 (mL/min/1.73 m^2^).

### 4.4. Acute Kidney Injury (AKI)

For the purposes of this study, renal function had to stable on the third postoperative day. Renal function was considered unstable in patients with AKI according to 2012 KDIGO guidelines. Ased on the 2012 KDIGO criteria, AKI was determined by the first KDIGO criterion, which is an increase in serum creatinine of ≥50% from baseline or ≥26.5 µmol/L within 48 h [59]. The lowest serum creatinine value from admission to discharge was used as baseline. Two time intervals were used to determine creatinine change within 48 h: first to second postoperative day, and second to third postoperative day. Patients meeting this definition of AKI were excluded.

### 4.5. Medications

For all patients, we simulated the total daily doses of oral formulated ibuprofen, morphine, and gabapentin the participants would be prescribed based on their renal function according to the six eGFR equations. In patients with eGFR ≥ 90 mL/min/1.73 m^2^ standard doses of ibuprofen, morphine, and gabapentin, are 1600, 20, and 1200 mg, respectively.

In case of eGFR < 90 mL/min/1.73 m^2^ recommendations for dose reduction according to renal function were determined for each GFR equation by recommendations in Renbase^®^ according to renal function [47,63]. Renbase^®^ offers medication-specific dose adjustments for each stage of renal impairment. For ibuprofen, the dose recommendations are: 1600 mg for eGFR ≥ 60; 1200 mg for eGFR 30–59; and contraindicated for eGFR ≤ 29. For morphine, the dose recommendations are: 20 mg for eGFR ≥ 90; 15 mg for eGFR 60–89; 10 mg for eGFR 30–59; and contraindicated for eGFR ≤ 29. For gabapentin, the dose recommendations are: 1800 mg for eGFR ≥ 90; 900 mg for eGFR 60–89; 600 mg for eGFR 30–59; 300 mg for eGFR 16–29; and 150 mg for eGFR ≤ 15.

### 4.6. Statistical Analyses

A mixed linear model was used with patient ID modelled as a random effect and type of equation as fixed effect to evaluate differences in eGFR between the equations. Goodness of fit was checked by visual inspection of the following plots: histogram of residuals to inspect normal distribution, scatter plot of residuals versus predicted values to inspect variance homogeneity. To test the agreement between the five CKD stages calculated from the eGFR equations, a weighted kappa statistic (к) was used. A kappa statistic of 0.21–0.40 was considered fair agreement; 0.41–0.60 moderate agreement; 0.61–0.80 substantial agreement, and 0.81–1.00 almost perfect agreement [64]. To test whether the dosage of ibuprofen, morphine, and gabapentin was dependent on eGFR equation, a Wilcoxon Matched-Pairs Signed Ranks Test was performed. For all statistical tests, *p* ≤ 0.05 was considered statistically significant and data are presented as mean and standard deviation (SD). All calculations and statistical analyses were performed in SAS Enterprise Guide 7.1.

## 5. Conclusions

In the current study, significant differences in eGFR based on the BIS, CG, FAS, LMR, and MDRD equations were found when compared with the CKD-EPI equation in elderly acute hip fracture patients. The CG, BIS, FAS, and LMR equations estimated GFR to be lower than CKD-EPI, while the MDRD equation estimated GFR to be higher. The BIS, FAS, and LMR estimates had a high level of agreement. Choice of eGFR equation in elderly acute hip fracture patients would have a significant impact on the dosing of ibuprofen, morphine, and gabapentin according to the renal function. Future research should focus on which eGFR equations and biomarkers are most accurate in the elderly population, and it is recommended that clinicians take caution when using creatinine-based equations to estimate the dose of renally excreted analgesics to elderly patients.

## Figures and Tables

**Table 1:** Demographic data (n = 118), median values with range.

Variable	Value
Female sex, n (%)	80 (67.8)
Age (years)	82.6 (70.1–100.8)
Actual body weight (kg)	63.0 (32.0–98.0)
Height (cm)	167 (144–191)
Body Mass Index (kg/m^2^)	22.4 (14.2–33.3)
Body Mass Index (kg/m^2^) ≤ 18.5, n (%)	14 (11.9)
Body Mass Index (kg/m^2^) > 30.0, n (%)	3 (2.50)
Body surface area (m^2^)	1.71 (1.19–2.19)
Body surface area > 1.9 m^2^, n (%)	25 (21.2)
Body surface area < 1.6 m^2^, n (%)	40 (33.9)
Serum creatinine (µmol/L)	71.0 (25.0–430)
*Comorbidities and medication*	
Hypertension, n (%)	56 (47.5)
Osteoporosis, n (%)	34 (28.8)
Dementia, n (%)	21 (17.8)
Ischemic heart disease, n (%)	19 (16.1)
Diabetes, n (%)	18 (15.2)
Number of medication at admission	6 (0–21)

**Table 2:** Estimated eGFR (mL/min/1.73 m^2^) and mean difference in eGFR values between the CKD-EPI standard equation and the five alternative eGFR equations (n = 118). *p*-values illustrate differences compared with the CKD-EPI equation.

Source of Equation	eGFR (Mean ± SD)	Estimated Difference in eGFR	95% Cl	*p*-Value
CKD-EPI	67.3 ± 22.3	-	-	-
BIS	59.1 ± 21.3	−8.2	−10.0–−6.4	<0.0001
CG	56.9 ± 25.7	−10.4	−12.2–−8.6	<0.0001
FAS	60.3 ± 24.6	−7.0	−8.8–−5.2	<0.0001
LMR	58.9 ± 20.1	−8.4	−10.2–−6.6	<0.0001
MDRD	79.1 ± 33.6	+11.8	10.0–13.6	<0.0001

*eGFR* estimated Glomerular Filtration Rate, *CKD-EPI* Chronic Kidney Disease Epidemiology Collaboration, *BIS* Berlin Initiative Study, *CG* Cockcroft-Gault, *FAS* Full Age Spectrum, *LMR* Lund-Malmö revised, *MDRD* Modification of Diet in Renal Disease.

**Table 3:** Classification of chronic kidney disease stages based on the six different creatinine-based eGFR equations. Data are represented as the number (percentage) of participants in each chronic kidney disease stage (n = 118).

Source of Equation	CKD I eGFR ≥ 90	CKD II eGFR 60–89	CKD III eGFR 30–59	CKD IV eGFR 15–29	CKD V eGFR < 15
CKD-EPI	15 (12.7)	64 (54.2)	31 (26.3)	6 (5.1)	2 (1.7)
BIS	7 (5.9)	46 (39.0)	58 (49.2)	5 (4.2)	2 (1.7)
CG	6 (5.1)	42 (35.6)	56 (47.4)	12 (10.2)	2 (1.7)
FAS	10 (8.5)	43 (36.4)	54 (45.8)	9 (7.6)	2 (1.7)
LMR	4 (3.4)	55 (46.6)	47 (39.8)	10 (8.5)	2 (1.7)
MDRD	41 (34.8)	45 (38.1)	25 (21.2)	5 (4.2)	2 (1.7)

*CKD* Chronic Kidney Disease classification, *eGFR* estimated Glomerular Filtration Rate, *CKD-EPI* Chronic Kidney Disease Epidemiology Collaboration, *BIS* Berlin Initiative Study, *CG* Cockcroft-Gault, *FAS* Full Age Spectrum, *LMR* Lund-Malmö revised, *MDRD* Modification of Diet in Renal Disease.

**Table 4:** The agreement of chronic kidney disease (CKD) stage among the six different creatinine-based eGFR equations in relative values. Weighted kappa coefficient (95% Cl), (number of patients with agreement in CKD stage; percentage patients with agreement in CKD stage).

	BIS	CG	FAS	LMR	MDRD
**CKD-EPI**	0.65 (0.54–0.76) (83; 70.4%)	0.57 (0.46–0.68) (75; 63.6%)	0.68 (0.57–0.78) (84; 71.2%)	0.65 (0.54–0.75) (83; 70.4%)	0.70 (0.60–0.79) (84; 71.2%)
**BIS**		0.78 (0.68–0.87) (97; 76.2%)	0.93 (0.87–0.98) (111; 94.0%)	0.85 (0.77–0.92) (104; 88.1%)	0.45 (0.34–0.56) (59; 50.0%)
**CG**			0.82 (0.74–0.90) (100; 84.7%)	0.80 (0.71–0.89) (100; 84.7%)	0.38 (0.28–0.49) (60; 50.8%)
**FAS**				0.87 (0.80–0.94) (105; 89.0%)	0.46 (0.36–0.56) (50; 42.4%)
**LMR**					0.44 (0.34–0.54) (49; 41.5%)

*CKD-EPI* Chronic Kidney Disease Epidemiology Collaboration, *BIS* Berlin Initiative Study, *CG* Cockcroft-Gault, *FAS* Full Age Spectrum, *LMR* Lund-Malmö revised, *MDRD* Modification of Diet in Renal Disease.

**Table 5:** The agreement of simulated recommended doses of ibuprofen, morphine, and gabapentin among the six different creatinine-based eGFR equations in relative values (n = 118). Number of patients with agreement in dosage (number of patients where y doses higher than x/number of patients where y doses lower than x). Ibuprofen is marked with cursive font. Morphine and gabapentin are marked with bold font.

	BIS	CG	FAS	LMR	MDRD
**CKD-EPI**	*91 (26/1)* **83 (34/1)**	*81 (37/0)* **72 (46/0)**	*89 (29/0)* **84 (34/0)**	*94 (24/0)* **83 (35/0)**	*110 (0/8)* **84 (0/34)**
**BIS**		*100 (15/3)* **97 (17/4)**	*114 (4/0)* **111 (4/3)**	*107 (5/6)* **104 (6/8)**	*85 (0/33)* **51 (0/67)**
**CG**			*104 (3/11)* **100 (3/15)**	*101 (2/15)* **99 (4/15)**	*73 (0/45)* **41 (0/77)**
**FAS**				*111 (1/6)* **105 (7/6)**	*81 (0/37)* **50 (0/68)**
**LMR**					*86 (0/32)* **49 (0/69)**

*CKD-EPI* Chronic Kidney Disease Epidemiology Collaboration, *BIS* Berlin Initiative Study, *CG* Cockcroft-Gault, *FAS* Full Age Spectrum, *LMR* Lund-Malmö revised, *MDRD* Modification of Diet in Renal Disease.

## Appendix A

GFR estimating equations based on creatinine. For all the GFR estimating equations below, age is expressed in years and eGFR in mL/min/1.73 m^2^ body surface area. Ln = natural logarithm.

CKD-EPI equation [24]: Serum creatinine (SCr) is expressed in mg/dL, 141 × min(SCr/*ᴋ*,1)^α^ × max(SCr/*ᴋ*,1)^−1.209^ × 0.993^Age^ × 1.018 (if female) × 1.159 (if black), *ᴋ* is 0.7 for females and 0.9 for males, α is 0.329 for females and 0.411 for males, min is the minimum of SCr/ *ᴋ* and 1 and max is the maximum of SCr/*ᴋ* and 1.

BIS equation [29]: Serum creatinine (SCr) is expressed in mg/dL, 3.736 × SCr × Age^−0.95^ × 0.82 (if female).

CG equation [27]: Serum creatinine (SCr) is expressed in mg/dL, (140 − Age) × weight (kg)/72 × SCr × 0.85 (of female) × (1.73/BSA), body surface area was calculated using DuBois formula: 0.007184 × weight (kg)^0.425^ × height (cm)^0.725^.

FAS equation [33]: Serum creatinine (SCr) is expressed in mg/dL, 107.3/(SCr/QCr) × 0.988^(Age − 40)^ (if 40 years or older), QCr is 0.7 mg/dL for females and QCr is 0.9 mg/dL for males.

LMR equation [34]: Serum creatinine (SCr) is expressed in mmol/L, e^x^ = −0.0158 × Age + 0.438 × Ln(Age), x = if female and SCr < 150: x = 2.50 + 0.0121 x(150 − SCr), x = if female and SCr ≥ 150: x = 2.50 − 0.926 × In(SCr/150), x = if male and SCr < 180: x = 2.56 + 0.00968 × (180 − SCr), x = if male and SCr ≥ 180: x = 2.56 − 0.926 × In(SCr − 180).

MDRD equation [26]: Serum creatinine (SCr) is expressed in mg/dL, 175 × SCr^-1.154^ × Age^−0.203^ × 0.742 (if female).
